# Functions of Endothelial Cilia in the Regulation of Vascular Barriers

**DOI:** 10.3389/fcell.2020.00626

**Published:** 2020-07-09

**Authors:** Nan Ma, Jun Zhou

**Affiliations:** ^1^State Key Laboratory of Medicinal Chemical Biology, College of Life Sciences, Nankai University, Tianjin, China; ^2^Shandong Provincial Key Laboratory of Animal Resistance Biology, Collaborative Innovation Center of Cell Biology in Universities of Shandong, College of Life Sciences, Institute of Biomedical Sciences, Shandong Normal University, Jinan, China

**Keywords:** vascular barrier, endothelial cell, primary cilium, signaling pathway, disease

## Abstract

The vascular barrier between blood and tissues is a highly selective structure that is essential to maintain tissue homeostasis. Defects in the vascular barrier lead to a variety of cardiovascular diseases. The maintenance of vascular barriers is largely dependent on endothelial cells, but the precise mechanisms remain elusive. Recent studies reveal that primary cilia, microtubule-based structures that protrude from the surface of endothelial cells, play a critical role in the regulation of vascular barriers. Herein, we discuss recent advances on ciliary functions in the vascular barrier and suggest that ciliary signaling pathways might be targeted to modulate the vascular barrier.

## Introduction

Blood vessels are found throughout the body to deliver oxygen and nutrients to body tissues, and remove metabolic waste and carbon dioxide from tissues. In some organs in mammals, there are distinct boundaries between blood and tissues. These barriers separate blood from tissues and are composed of endothelial cells, pericytes, other vascular mural cells, and intercellular connections. In contrast to the common vascular barriers, there are extremely strict vascular barriers in some important organs, such as the blood-brain barrier (Chow and Gu, 2015; Yang Z. et al., 2019), the blood-retinal barrier (Diaz-Coranguez et al., 2017; Naylor et al., 2019), and the blood-testis barrier (Mruk and Cheng, 2015). Compared with the common vascular barriers, these barriers involve some special cells and intercellular connections. For example, 85% of the surface area of the brain capillary wall is surrounded by the astrocytic end-feet, and in the testicle, the supporting cells adjacent to the basement membrane of spermatogenic tubules are connected in a special structure (Gerber et al., 2016), which makes them less penetrating. The vascular barrier controls the exchange of molecules and ions between blood and tissues, prevents harmful substances from entering the tissue and causing damage, and maintains a stable internal tissue environment. In addition, in some tissues, the vascular barrier gives them immune privileges to avoid the occurrence of autoimmunity. However, once these vital organs have lesions, treatment is challenging as the vascular barrier prevents drugs from targeting the relevant tissues, making them ineffective (Pardridge, 2012).

The vascular barrier has been shown to be regulated by a number of signaling pathways, such as Mfsd2a, lipolysis stimulated lipoprotein receptor, angiopoietin-2, Wnt, and Norrin pathways (Liebner et al., 2008; Stenman et al., 2008; Yen et al., 2008; Daneman et al., 2009; Gao et al., 2010; Ye et al., 2010; Rangasamy et al., 2011; Ben-Zvi et al., 2014; Yu et al., 2017). In addition, the intestinal flora has been reported to affect the vascular barrier (Braniste et al., 2014; Al-Asmakh and Hedin, 2015). Some of the signaling mechanisms regulate initial angiogenesis, while others maintain the mature vascular barrier. Therefore, it is important to understand the molecular mechanisms that maintain vascular barriers and to develop new medicines to regulate the barriers. Recent evidence suggests that the primary cilia of endothelial cells play a critical role in regulating vascular barriers. In this review, we describe the functions of endothelial cilia in the development and maintenance of vascular barriers and discuss the potential of targeting cilium-related molecules to modulate the barriers.

## Vascular Endothelial Cilia

**FIGURE 1:**
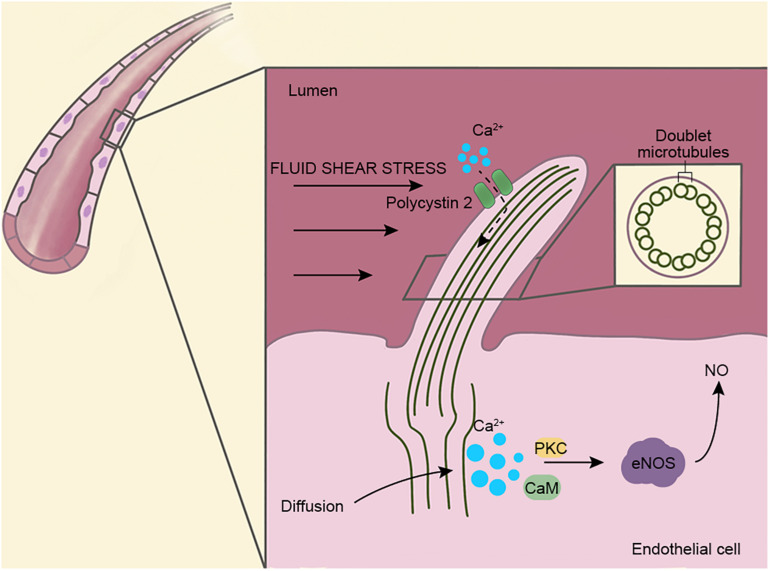
Structure and function of vascular endothelial cilia. Primary cilia are present on the surfaces of vascular endothelial cells and extend into the lumen. Cilia are used as mechanoreceptors and signaling centers to regulate vascular functions. The ciliary axenome is composed of nine pairs of doublet microtubules and surrounded by a ciliary membrane, where several transport proteins and ion channels are present. Fluid shear stress is known to open Ca^2+^ channels on cilia (dotted line), but recent studies show that Ca^2+^ originates in the cytoplasm instead of cilia. Via the actions of protein kinase C (PKC) and calmodulin (CaM), endothelial nitric oxide synthase (eNOS) is activated and leads to the upregulation of NO, which dilates blood vessels and prevents vascular rupture caused by excessive blood flow.

Vascular endothelial cells play an important role in vascular barrier function. Recent studies demonstrate that primary cilia are present on vascular endothelial cells with typical “9 + 0” axonemes (Figure 1). Primary cilia are microtubule-based organelles, which are ubiquitous in mammals (Satir and Christensen, 2008; Yang et al., 2014; Yang Y. et al., 2019; Yu et al., 2016, 2019; Ran et al., 2020). Cilia are mainly composed of the basal body, the axoneme, the ciliary matrix, and the ciliary membrane. Transport proteins, ion channels, and receptors are embedded in the membrane (Satir et al., 2010). The basal body is localized at the base of the cilium and is derived from the mother centriole of the centrosome. In addition, the basal body consists of various appendages, as well as the pericentriolar material. The formation and maintenance of cilia depend on intraflagellar transport (IFT) proteins, motor proteins, and structural components.

The primary cilia on the vascular endothelial cell extend into the lumen of the blood vessel and act as sensors and transmit extracellular signals into the cell (Pala et al., 2017). In addition, endothelial cilia regulate blood vessel function through blood flow sensing, cell migration, and calcium and nitric oxide (NO) signaling (Nauli et al., 2008; Jones et al., 2012). Ca^2+^ is an important second messenger that regulates many signaling pathways and regulates vasoconstriction and vasodilation through NO in vascular endothelial cells. A number of previous studies have shown that fluid shear stress triggers ciliary calcium signaling, and this process is mediated by polycystin 2 (PC2) (Jin et al., 2014). Recently, however, Delling et al. (2016) have proposed the opposite, suggesting that Ca^2+^ are produced in the cytoplasm and rapidly diffuse into the cilia and that the primary cilia may not be involved in the influx of Ca^2+^ (Figure 1). Thus, the source of Ca^2+^ in endothelial cells needs to be investigated further.

Different blood flow or velocity has different effects on endothelial cells, resulting in changes in blood vessel phenotypes and functions, a process called hemodynamic stimulation (Chistiakov et al., 2017). The primary cilia of endothelial cells appear in the early stages of angiogenesis, indicating that cilia are related to early angiogenesis. Interestingly, this functionality does not depend on hemodynamic stimulation (Eisa-Beygi et al., 2018). This is different from the role of cilia in regulating blood vessels by blood flow stimulation after angiogenesis. In addition, the cilia of vascular endothelial cells can detect the extracellular pH and regulate related pathways to maintain the acid-base homeostasis of endothelial cells (Atkinson et al., 2019). Damage to the primary cilia of endothelial cells causes several vascular diseases (Mohieldin et al., 2016; Luu et al., 2018). The recent evidence on the correlation between endothelial cilia and the vascular barrier suggests that endothelial cilia may be a potential therapeutic target for vascular barrier-related diseases.

## Ciliary Functions in the Vascular Barrier

### IFT Proteins

Intraflagellar transport is a two-way transport system located between axonemal microtubules and ciliary membrane. It is the main transport system of cilia, and also transports proteins during cilium-related signaling, such as Hedgehog (HH) and Wnt signaling (Goetz and Anderson, 2010). The model proposed by Haycraft et al. (2005) suggests that IFT is indirectly involved in HH signal transduction, and that the absence of IFT leads to the failure of GLI protein processing, thus affecting the downstream signal transduction of HH. However, the function of cilia in Wnt signaling is controversial. Other studies show normal Wnt signaling in mouse mutants that lack cilia due to *Ift172* mutation (Eggenschwiler and Anderson, 2007). Normal Wnt signaling has also been shown in zebrafish mutants that lack all cilia, suggesting that cilia are not necessary for Wnt signaling (Huang and Schier, 2009). Therefore, whether cilia play a role in Wnt signaling requires further study. In addition, proteins in the cilia can be transported back to the cytoplasm through IFT to ensure the protein balance in the cilia (Lechtreck et al., 2013). IFT is essential for cilium assembly, maintenance, and perception. The loss of IFT leads to abnormal cilia. A zebrafish embryo model study demonstrates that cilia are present in the vascular endothelial cells of the zebrafish brain, and after IFT protein mutations, the probability of intracranial hemorrhage increases compared with the control group. However, no changes in blood vessel morphology and cell connections are observed. After re-expressing the relevant IFT protein, the bleeding phenotype improves significantly (Kallakuri et al., 2015).

In the mouse embryo model, *Ift172* and *Ift122* mutants show cranial neural tube defects and bleeding (Cortellino et al., 2009; Gorivodsky et al., 2009), but the molecular mechanism of the bleeding phenotype is still unclear. Another study has evaluated the permeability of endothelial cells from *Ift88* mutant mice with polycystic kidney disease (PKD). Compared with wild-type cells, *Ift88* mutant endothelial cells have reduced stress fibers and focal adhesion, and higher permeability to dextran, indicating that cilium defects cause damage to the vascular barrier. The expression of heat shock protein 27 in *Ift88* mutant endothelial cells is inhibited by approximately 90%, and the phosphorylation level of its downstream target, focal adhesion kinase, is significantly reduced (Jones et al., 2012). These studies indicate that IFT proteins play a potential role in the vascular barrier.

### Polycystin Proteins

In addition to IFT proteins, polycystin 1 (PC1), is another protein related to cilia that show a correlation with the vascular barrier. PC1 usually forms a channel-receptor complex with PC2. These transmembrane proteins regulate cell activity by detecting external stimuli and transmitting calcium-mediated signals (Qian et al., 1997). PC1 is expressed in endothelial cells and smooth muscle cells and is involved in cell-matrix connectivity. *PKD1* (the gene encoding PC1) mutations cause autosomal dominant PKD, and vascular abnormalities are usually observed in these patients. Due to vascular leakage and hemorrhage, PKD1 knockout mice are embryonic lethal at embryonic day 15.5 (Kim et al., 2000). However, the mechanism is not clear and further studies are required. In zebrafish embryos, *PKD2* (the gene encoding PC2) mutations demonstrate no bleeding phenotype (Kallakuri et al., 2015), suggesting that PC1, but not PC2, plays a major role in the maintenance of the vascular barrier.

### HH Signaling

**FIGURE 2:**
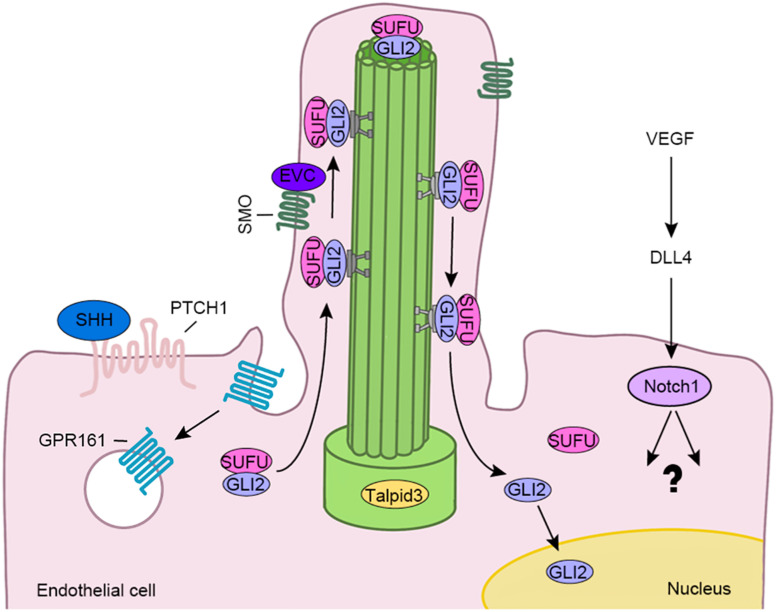
The endothelial cilium regulates various signaling pathways involved in the vascular barrier, such as HH and Notch pathways. The binding of SHH to PTCH1 leads to SMO activation and enrichment at the cilium. GPR161, the negative regulator of the HH pathway, then is internalized from the cilium. Subsequently, the GLI2/SUFU complex is transported to the ciliary tip by the IFT complex. After the complex is dissociated, GLI2 is activated and translocated to the nucleus. In addition, vascular endothelial growth factor (VEGF) induces the expression of delta like canonical Notch ligand 4 (DLL4), which then binds to Notch1 to activate downstream events.

Hedgehog signaling is critical for vascular development, maturity and integrity (Chapouly et al., 2019), and is one of the signaling pathways closely related to the function of the primary cilia (Chow and Gu, 2015). HH signaling elements, including patched 1 (PTCH1), G protein-coupled receptor 161 (GPR161), smoothened (SMO), suppressor of fused protein (SUFU), GLI family zinc finger 2 (GLI2), and GLI3, are located on primary cilia (Figure 2) (Bangs and Anderson, 2017). HH is a lipoprotein morphogenetic factor that participates in the development of vertebrate tissues and the maintenance of stem cell homeostasis, both essential for central nervous system development (Briscoe and Therond, 2013). Sonic HH (SHH) signaling, one of the HH signaling pathways, plays an important role to maintain the blood-brain barrier in embryos and adults. Specifically, astrocytes that comprise the blood-brain barrier structure, secrete SHH, and promote the expression of GLI1 and Sox18 in endothelial cells to upregulate the expression of occludin and claudin-5 and reduce endothelial cell permeability (Alvarez et al., 2011). Using zinc-finger nuclease-mediated or morpholino oligo-mediated mutagenesis to destroy genes encoding ciliary proteins, such as IFT proteins, Talpid3, and Dzip1/Iguana, Bangs and Anderson show characteristics of SHH deficiency (Bangs and Anderson, 2017). Together, these results indicate that cilia play a critical role in HH signaling.

The zebrafish model study shows that when the HH signaling is inhibited due to ciliary defects, the possibility of intracranial hemorrhage increases. Conversely, activation of this pathway reduces the risk of cerebral hemorrhage (Kallakuri et al., 2015), indicating that endothelial cilia may regulate vascular integrity through the HH signaling pathway. Furthermore, Talpid3 is an essential protein in ciliogenesis, and its mutation in zebrafish causes abnormal HH signaling. Embryos exhibit cerebral hemorrhage symptoms (Ben et al., 2011), which confirms the connection between cilia and vascular integrity. HH signaling is also related to retinal angiogenesis, because treatment with cyclopamine, an HH signal inhibitor, inhibits retinal angiogenesis. HH signaling is required for survival of retinal endothelial cells and pericytes (Surace et al., 2006).

### Notch Signaling

The Notch pathway is the main signaling pathway that coordinates angiogenesis with angiogenin signaling and cell metabolism (Tetzlaff and Fischer, 2018). Notch signaling promotes the formation of functional blood vessels by regulating the differentiation of endothelial cells. Recent studies show that cilium-specific genes are required for the development of hematopoietic stem cells and progenitor cells in endothelial cells during zebrafish embryogenesis, which is primarily mediated by Notch signaling (Liu et al., 2019). Disassembly of cilia in *Ift88* knockout mice could reduce Notch activity, resulting in thickening of corneal epithelial cells (Grisanti et al., 2016). Limited data about the function of Notch signaling in the formation and maintenance of the vascular barrier is available. Cilium-related proteins may regulate the vascular barrier function through various signaling pathways. A better understanding of the upstream effector molecules and downstream targets of these signaling pathways will help elucidate the molecular mechanisms regulating vascular barriers (Figure 2).

## Targeting Cilia to Modulate the Vascular Barrier

Due to the high selectivity of the vascular barrier, it is challenging to deliver drugs to certain tissues. It is therefore important to modulate the vascular barrier. Some solutions have been documented. For example, in the blood-brain barrier, the use of mannitol or focused ultrasound can temporarily open the junction between endothelial cells, so that molecules unable to pass through the barrier can temporarily enter the brain tissue. However, simultaneously, substances such as plasma proteins in blood vessels that may cause harm to brain tissue, are able to enter (Rodriguez et al., 2015). *In vitro* studies show that glucocorticoids improve the integrity of the blood-brain barrier in patients with multiple sclerosis, but this method alone can cause serious adverse reactions (Blecharz et al., 2010). Stimulants such as histamine, atrial natriuretic peptide, or thrombin increase the permeability of blood vessels (Konstantoulaki et al., 2003; Ashina et al., 2015).

Maintenance of the vascular barrier depends on the regulation of various signaling pathways. Cilia play an important role in some of these signaling pathways, especially in HH signaling, and indirectly regulates the formation and maintenance of the vascular barrier. A number of studies have shown that there is a certain relationship between cilia and the integrity of the vascular barrier. In addition, the target of certain drugs, such as cytochalasin D, is part of the signaling pathway regulated by cilia (Kim et al., 2010). Thus, targeting cilia (e.g., delivery of cilium-related genes with adenovirus vectors) is a potential strategy for treating diseases related to the vascular barrier (Kotterman and Schaffer, 2014), which requires a joint effort among clinicians and scientists.

## Conclusion and Perspectives

The vascular barrier plays a key role to maintain tissue homeostasis, while cilia are involved in the generation and maintenance of the vascular barrier. Although more evidence is available, several aspects have not been resolved. For example, it is not clear whether cilia play different roles in different tissues and how cilia regulate various parts of the vascular barrier through different signaling pathways. The molecules responsible for regulating the formation and maturation of the vascular barrier, the method used to assess the vascular barrier and how to target cilia to regulate the vascular barrier with treatments still need to be investigated. Further research will provide new strategies for the treatment of vascular diseases caused by ciliary defects.

Destruction of cilium-related proteins can damage the vascular barrier, which may be mediated by HH, Notch, Wnt, and other signaling pathways. It is possible that cilium defects cause damage to intercellular junctions and paracellular transport, such as tight junctions and adherens junctions, which are important in vascular barriers (Xie et al., 2017; Dong et al., 2020). Alternatively, cilium defects may cause blood vessels to inhibit the recruitment of vascular mural cells, such as pericytes and smooth muscle cells, leading to damage to the vascular barrier (Chen et al., 2017). Knowledge of the link between cilium defects and human diseases continues to expand, and vascular dysfunction is present in some ciliary diseases. Maintenance of the vascular barrier involves several connexins, transmembrane proteins, and related signaling pathways. Defects in one of these proteins may lead to the destruction of the vascular barrier, and the same protein may play a role in ciliary signaling. In addition, compensation mechanisms in some signaling pathways may be present, which makes it difficult to study the specific role of cilia in the regulation of vascular barriers. At present, the mechanisms of how cilia are involved in maintaining vascular barriers remain elusive. A better understanding of this question will be undoubtedly helpful to the treatment of vascular barrier-related diseases.

## Author Contributions

NM wrote the manuscript and drew the figures. JZ conceived the study and revised the manuscript. Both authors read and approved the final version of the manuscript.

## Conflict of Interest

The authors declare that the research was conducted in the absence of any commercial or financial relationships that could be construed as a potential conflict of interest.
